# Ultraviolet Irradiation of Skin Alters the Faecal Microbiome Independently of Vitamin D in Mice

**DOI:** 10.3390/nu10081069

**Published:** 2018-08-11

**Authors:** Simon Ghaly, Nadeem O. Kaakoush, Frances Lloyd, Lavinia Gordon, Cynthia Forest, Ian C. Lawrance, Prue H. Hart

**Affiliations:** 1 Telethon Kids Institute, The University of Western Australia, Perth, WA 6008, Australia; prue.hart@telethonkids.org.au; 2 School of Medicine and Pharmacology, The University of Western Australia, Perth, WA 6009, Australia; frances.p.lloyd8@gmail.com (F.L.); ian.lawrance@uwa.edu.au (I.C.L.); 3 Department of Gastroenterology and Hepatology, St. Vincent’s Hospital, Sydney, NSW 2010, Australia; 4 School of Medical Sciences, UNSW Sydney, Kensington, NSW 2033, Australia; n.kaakoush@unsw.edu.au; 5 Australian Genome Research Facility, The Walter and Eliza Hall Institute, Parkville, VIC 3052, Australia; Lavinia.Gordon@agrf.org.au; 6 Department of Anatomical Pathology, PathWest, Fiona Stanley Hospital, Murdoch, WA 6150, Australia; Cindy.Forrest@health.wa.gov.au; 7 Centre for Inflammatory Bowel Disease, St. John of God Hospital, Subiaco, WA 6008, Australia

**Keywords:** ultraviolet radiation, microbiome, vitamin D, inflammatory bowel disease

## Abstract

Reduced sunlight exposure has been associated with an increased incidence of Crohn’s disease and ulcerative colitis. The effect of ultraviolet radiation (UVR) on the faecal microbiome and susceptibility to colitis has not been explored. C57Bl/6 female mice were fed three different vitamin D-containing diets for 24 days before half of the mice in each group were UV-irradiated (1 kJ/m^2^) for each of four days, followed by twice-weekly irradiation of shaved dorsal skin for 35 days. Faecal DNA was extracted and high-throughput sequencing of the 16S RNA gene performed. UV irradiation of skin was associated with a significant change in the beta-diversity of faeces compared to nonirradiated mice, independently of vitamin D. Specifically, members of phylum Firmicutes, including *Coprococcus*, were enriched, whereas members of phylum Bacteroidetes, such as Bacteroidales, were depleted. Expression of colonic *CYP27B1* increased by four-fold and *IL1β* decreased by five-fold, suggesting a UVR-induced anti-inflammatory effect. UV-irradiated mice, however, were not protected against colitis induced by dextran sodium sulfate (DSS), although distinct faecal microbiome differences were documented post-DSS between UV-irradiated and nonirradiated mice. Thus, skin exposure to UVR alters the faecal microbiome, and further investigations to explore the implications of this in health and disease are warranted.

## 1. Introduction

The health of the gastrointestinal tract is dependent on the bidirectional interaction between gut microbial antigens and the intestinal immune system to maintain homeostasis or “physiological inflammation”. In inflammatory bowel disease (IBD), Crohn’s disease (CD), and ulcerative colitis (UC), there is a dysregulated immune response against luminal antigens leading to uncontrolled inflammation. It remains unclear if the primary problem is the dysregulated immune response or change in luminal antigens, or as is most likely the case, a combination of both. Ultraviolet (UV) irradiation of skin has both vitamin D-dependent and -independent effects on systemic immunity, and interestingly, IBD is increased in areas of low sun exposure and higher latitudes [1,2,3,4,5].

Diet-induced vitamin D deficiency is associated with altered faecal microbial composition in C57Bl/6 mice, with an increase in the relative quantities of Bacteroidetes, Firmicutes, Actinobacteria, and Gammaproteobacteria in naïve, noncolitic mice [6]. This group has recently demonstrated reduced global β-diversity in faeces from mice fed diets with high vitamin D content compared to no vitamin D, as well as 40 microbial taxa that were significantly different between the groups [7]. The vitamin D-independent effect of skin exposure to UV radiation (UVR) on the composition of intestinal microbiota remains unexplored and is important in understanding whether sun exposure is equivalent to a vitamin D tablet in its impact in both health and disease.

The vitamin D-independent pathways by which UVR may suppress immunity have yet to be fully elucidated; however, a number of mechanisms have been proposed and reviewed elsewhere [8,9]. Two studies have examined the effect of phototherapy in an animal model of IBD using oral dextran sodium sulfate (DSS), which causes a chemical injury to the gastrointestinal tract that is then repaired by innate immune mechanisms [10]. These studies reported a reduction in disease severity with light therapy, but the findings are limited by the subjective measures of colitis severity, the small numbers of mice used, and most importantly, the lack of definition and consistency of the light sources used.

In this current study, the effect of UVR on the faecal microbiome was explored in the setting of high dietary vitamin D (D++), vitamin D sufficiency (D+), and vitamin D deficiency (D−). Furthermore, the effect of UVR on the severity of DSS colitis was studied.

## 2. Materials and Methods

### 2.1. Mice and Diets

Female 6-week-old C57Bl/6 mice were fed semipure diets containing high levels of vitamin D (SF14-069, Specialty Feeds, Perth, Western Australia, 10,000 IU/kg vitamin D_3_, 0.5% calcium), moderate levels of vitamin D to maintain vitamin D sufficiency similar to standard chow (SF05-34, Specialty Feeds, 2280 IU/kg vitamin D_3_, 1% calcium), or no vitamin D to induce vitamin D deficiency (SF05-033, Specialty Feeds, 0 IU/kg vitamin D_3_, 2% calcium). Female mice were used as we have previously shown that acute erythemal or short-term suberythemal UVR does not increase 25(OH)D_3_ levels in male mice [11,12]. All experiments were performed according to the ethical guidelines of the National Health and Medical Research Council of Australia with the approval from the Telethon Kids Institute Animal Ethics Committee (AEC #276). Mice were purchased from the Animal Resources Centre, Western Australia.

Mice were housed under perspex-filtered fluorescent lighting, which emitted no detectable UVB radiation as measured using a UV radiometer (UVX Digital Radiometer, Ultraviolet Products Inc., Upland, CA, USA).

### 2.2. UV Radiation

A bank of six 40 W lamps (Philips TL UV-B, Eindhoven, The Netherlands) emitting broadband UVR, 250–360 nm, with 65% of the output in the UVB range (280–315 nm), was used to irradiate mice and to deliver 1 kJ/m^2^ of UVR onto clean-shaven 8 cm^2^ dorsal skin. This dose of UVR is approximately 50% of the minimal erythemal dose for C57Bl/6 mice, i.e., 50% of the lowest amount of UVR causing just-perceptible erythema after 24 h. A new sheet of polyvinyl chloride (PVC) plastic film (0.22 mm) was taped to the top of each perspex cage immediately before irradiation to screen wavelengths <290 nm. Sunlamps were held 20 cm above the cages. UV irradiation was performed consistently between the hours of 08:00 and 11:00 h. The dorsal skin of mice not treated with UVR was also shaved and the mice were handled in an identical fashion to UV-irradiated mice, including being placed in the UV irradiation room for the same duration.

### 2.3. Faecal Microbiota Analysis

The faecal microbiome was analysed by sequencing the V3–V4 segment of the 16S ribosomal RNA (rRNA) gene using Illumina MiSeq chemistry. Faecal pellets were collected and stored at −20 °C for up to six months. Bacterial DNA was extracted using the PowerSoil^®^ DNA isolation kit according to the manufacturer’s instructions (MO BIO Laboratories, Carlsbad, CA, USA). PCR amplification (341F/806F primer pair) and sequencing was performed by the Australian Genome Research Facility on the Illumina MiSeq (San Diego, CA, USA) with 2 × 300 bp paired-end chemistry. DNA extraction controls were used to account for any contaminants. Paired-end reads were assembled by aligning the forward and reverse reads using PEAR (version 0.9.5) [13]. Primers were trimmed using Seqtk (version 1.0) [14]. Trimmed sequences were processed using Quantitative Insights into Microbial Ecology (QIIME 1.8) [15] USEARCH (version 7.1.1090) [16,17] and UPARSE [17] software. Using QIIME, taxonomy was assigned using Greengenes database (Version 13_8, 2013) [18].

Microbiome statistical analysis was undertaken using the programming language R, specifically the *phyloseq* and *edgeR* packages available through Bioconductor, a project providing tools for the analysis and comprehension of high-throughput genomic DNA. The biom file, operating taxonomy unit (OTU) table, taxonomic assignments, and associated sample data were imported into R to create a *phyloseq* object. For all beta-diversity analyses, OTUs for which the variance across all samples was very low were filtered out. For testing a single categorical experimental condition, exact tests for differences in the means between two groups of negative-binomially distributed counts were computed. Data were normalised using the run-length encoding (RLE) scaling factor method and dispersions estimated. The counts were extracted and ranked by *p* value, applying a false discovery rate cutoff of less than 0.001. LEfSe (linear discriminant analysis effect size) was used to identify differentially abundant microbial taxa [19].

### 2.4. Real-Time PCR

Messenger RNA was extracted from snap-frozen colon and kidney with cDNA synthesised and real-time assays performed as previously described [20]. Real-time PCR primers were *CYP27B1* cat # 301447280210/0&1, *VDR* cat# KSPQ12012G (Sigma-Aldrich, St. Louis, MO, USA), *CAMP* cat# QT00241003, *IL-1β* cat# QT01048355 (Qiagen, Hilden, Germany). Housekeeping genes used were TATA-box-binding protein for colonic tissue (Sigma-Aldrich) and elongation factor 1α for kidney tissue (Sigma-Aldrich). Quantitect SYBRGreen was used for qPCR on the AB17900HT instrument. Fold-change was determined by using the 2^−∆∆Ct^ method.

### 2.5. Colitis Model

After 28 days on respective diets, with or without UVR exposure on days 24–28, half of the mice underwent treatment to induce colitis by the addition of DSS (3% (wt/vol) (MP Biomedicals LLC, OH)) to the drinking water for 6 days (Supplementary Figure S1). As the efficacy of DSS varies between batches, all experiments were conducted using the same batch [21]. In preliminary experiments, 3% DSS induced adequate colitis with peak weight loss ranging between 0.3% and 10.3% after 6 days of DSS treatment. Following induction of colitis, mice recovered over a period of 0–4 weeks without ongoing DSS treatment. Mouse body weight was assessed daily during DSS treatment and weekly during recovery. The experiment was repeated, with a total of 35 mice per group. Mice were sacrificed at days 35, 42, 49, and 63.

### 2.6. Murine Colonoscopy

A high-resolution mouse video endoscopic system was used to assess the level of colitis. All mice were colonoscoped after 6 days of DSS treatment and then at the time of sacrifice. Mice were anaesthetised using isofluorane unless the colonoscopy was being performed at the end-point, when ketamine 20 mg/mL and xylazine 2 mg/mL by intraperitoneal injection was used. All procedures were digitally recorded and then scored in a blinded fashion. The experimental endoscopy setup consisted of a miniature endoscope (1.9 mm outer diameter), a xenon light source, a triple chip camera, and an air pump (Karl Storz, Germany) to achieve regulated inflation of the mouse colon.

The severity of colitis was determined using the modified Murine Endoscopic Index of Colitis Severity (MEICS) [21,22]. The MEICS system consists of five parameters: thickening of the colon wall, changes of the normal vascular pattern, presence of fibrin, mucosal granularity, and stool consistency. Endoscopic grading was performed for each parameter (scored between 0 and 3) leading to a cumulative score of between 0 (no signs of inflammation) and 15 (endoscopic signs of severe inflammation). Healthy mice had a score of 0–3.

### 2.7. Histological Assessment of Colitis

Colons were removed with the rectum discarded as this has a different tissue fibro-structure. The distal 1 cm of colon was dissected, cleaned, formalin-fixed, and embedded in paraffin wax. Sections were stained with haemotoxylin and eosin (H&E). All H&E sections were assessed blindly by a specialist gastroenterological histopathologist (CF) according to the scoring system by Dieleman et al. [23]. In this scoring system, the severity and depth of inflammation as well as the level of crypt damage and regeneration are scored.

### 2.8. Measurement of Serum Metabolites 

At the time of sacrifice, blood was drawn by cardiac puncture. Levels of 25(OH)D_3_ were measured in the serum by liquid chromatography tandem mass spectroscopy (LC/MS/MS) [24]. Levels of 1,25(OH)_2_D_3_ were measured using IDS EIA ELISA kits (Immunodiagnostic Systems, Fountain Hills, AZ, USA) as described by the manufacturer.

Serum cytokines were measured using Bio-Plex Pro™ Mouse Cytokine 23-plex panel (Bio-Rad Laboratories, Hercules, CA, USA) as per the manufacturer’s instructions. The cytokines analysed included interleukin (IL)-1α, IL-1β, IL-2, IL-3, IL-4, IL-5, IL-6, IL-9, IL-10, IL-12 p40, IL-12 p70, IL-13, IL-17, eotaxin (CCL11), G-CSF, GM-CSF, IFN-γ, KC, MCP-1, MIP-1α, MIP-1β, RANTES, and TNF-α.

### 2.9. Statistical Analyses

Statistical significance was calculated using IBM^®^ SPSS^®^ Statistics Version 22 (IBM Corp. Armonk, NY, USA). All graphs and comparison of differences between groups were assessed using Student’s unpaired *t*-test or ANOVA with post-hoc least significant difference (LSD) analysis for multiple group analysis. Nonparametric data were analysed using Mann–Whitney *U* and Kruskall–Wallis testing.

## 3. Results

### 3.1. Microbiome Changes in UV-Irradiated Mice

**Figure 1:**
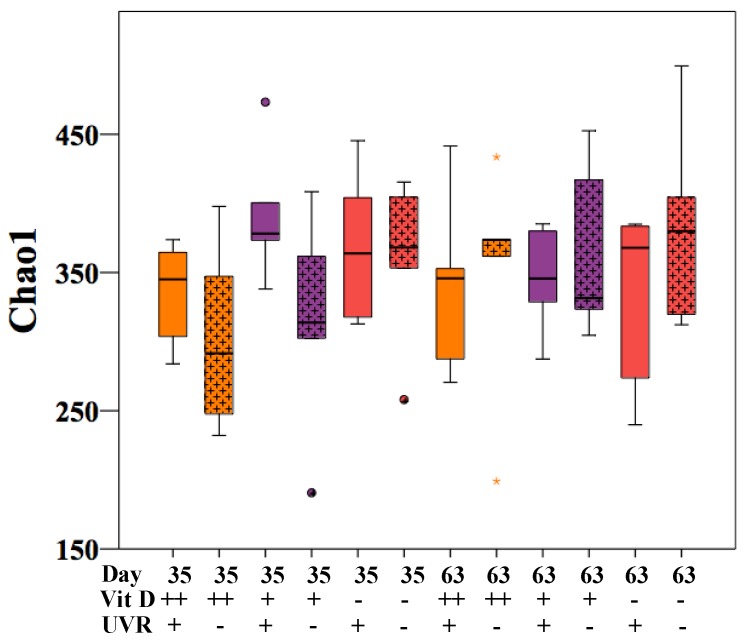
Alpha diversity of faecal samples. Faecal pellets were collected from UV-irradiated and nonirradiated mice that had been given either a high-dose (D++), standard-dose (D+), or no vitamin D (D−) diet. Mice were first established on diets for 24 days (days 0–24), then half were UV-irradiated daily with 1 kJ/m^2^ UVR for 4 consecutive days (days 24 to 28), followed by biweekly exposures. Samples were collected after 6 UV treatments (day 35) and at the end of follow-up (protocol day 63). After faecal DNA was extracted, the V3–V4 segment of 16S rRNA was sequenced using the Illumina MiSeq. Alpha diversity is represented by Chao1. *n* = 5/group. UVR = ultraviolet radiation.

After being established on the respective vitamin D diets for 24 days (days 0 to 24), half of the mice in each group received daily UVR, 1 kJ/m^2^, for four days (days 24 to 28), followed by twice-weekly treatment with 1 kJ/m^2^ UVR in an attempt to mimic physiological UVR exposure. Microbiota analysis was performed on 60 faecal samples, comprising five samples from each group (D++UVR+, D++UVR−, D+UVR+, D+UVR−, D-UVR+, D-UVR−) at day 35 and day 63. Nonmetric multidimensional scaling (NMDS) plot of the Bray–Curtis resemblance matrix following square-root transformation of relative abundance data showed an outlier control mouse in the D+UVR− group which was removed from all further analyses. Among mice irradiated with UV, there was no difference in alpha-diversity as measured by Chao1 in faecal samples harvested at both days 35 and 63, compared to the corresponding nonirradiated group (Figure 1).

The effects of UVR on the beta-diversity of individual vitamin D groups were examined at day 35 and day 63 by PERMANOVA, but no significant differences were seen (Table 1).

**Figure 2:**
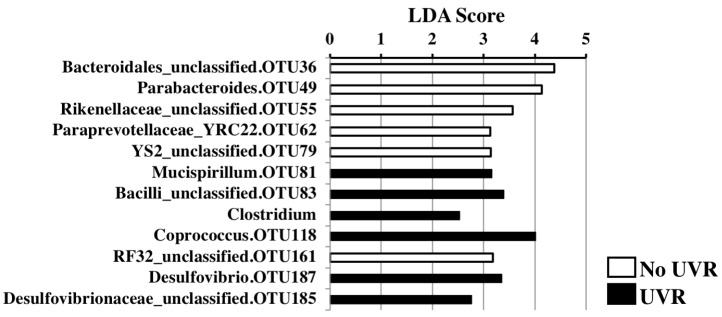
Faecal microbial composition of UV-irradiated vs nonirradiated mice. The effect of UV irradiation of skin on faecal microbial composition was examined using two-factor PERMANOVA controlling for vitamin D group. Linear discrimination analysis effect size (LDA score) was used to determine significant differences in relative abundance of individual taxa with UV treatment. Only taxa where a significant change (*p* < 0.05) was observed are illustrated. *n* = 44/group.

When all groups were analysed together using multifactor PERMANOVA (*n* = 60), there was a significant difference in overall beta-diversity with UV irradiation, independent of the effect of vitamin D (*t* = 1.7, *p* = 0.009, Perms = 999, degrees of freedom (df) = 53). Linear discriminant analysis (LDA) scores were calculated for individual taxa that significantly change in relative abundance with UVR compared to no UVR exposure (Figure 2). An enrichment of *Coprococcus* (LDA score 4.01, *p* = 0.04) and *Mucispirillum* (LDA score 3.17, *p* = 0.04) with UVR exposure was observed. Conversely, Bacteroidales were more abundant in the faeces from the nonirradiated group, including an unclassified species (OTU 36, LDA = 4.38, *p* = 0.02) and *Parabacteroides* (LDA = 4.13, *p* = 0.04).

### 3.2. Effect of UV Irradiation of Skin on Serum Vitamin D and Cytokine Levels

After being fed the vitamin D diets for 35 days (day 0 to day 35) and exposure to daily UVR between days 24 to 28 and biweekly irradiation thereafter, blood was collected by cardiac puncture at protocol day 35. There was a trend towards higher serum 25(OH)D_3_ levels in the D+ group with UV irradiation (*p* = 0.06), but not in the D++ group (Supplementary Figure S2). The vitamin D-deficient group (D−) had the lowest serum 25(OH)D_3_ levels and, as expected, their levels increased significantly with UVR exposure to levels comparable to the vitamin D-sufficient (D+) group (Supplementary Figure S2).

UV irradiation of the mice caused a decrease in circulating IL-17 levels among D+ mice that trended to statistical significance (*p* = 0.05) (Table 2). Similar changes were not seen among D++ or D− mice. IL-17 levels, however, were lower among mice exclusively deriving vitamin D from UVR (D−UVR+; mean ± SEM = 121.5 ± 12.0 pg/mL, *n* = 4) compared to those deriving vitamin D from diet (D+UVR−; 207.9 ± 72.7 pg/mL, *n* = 3; *p* = 0.07). Serum protein levels of interleukin (IL)-1β, TNF, IL-10, and IL-6 did not change with UVR exposure.

### 3.3. Effect of UV Irradiation on Colonic and Kidney Vitamin D Pathway Gene Expression

**Figure 3:**
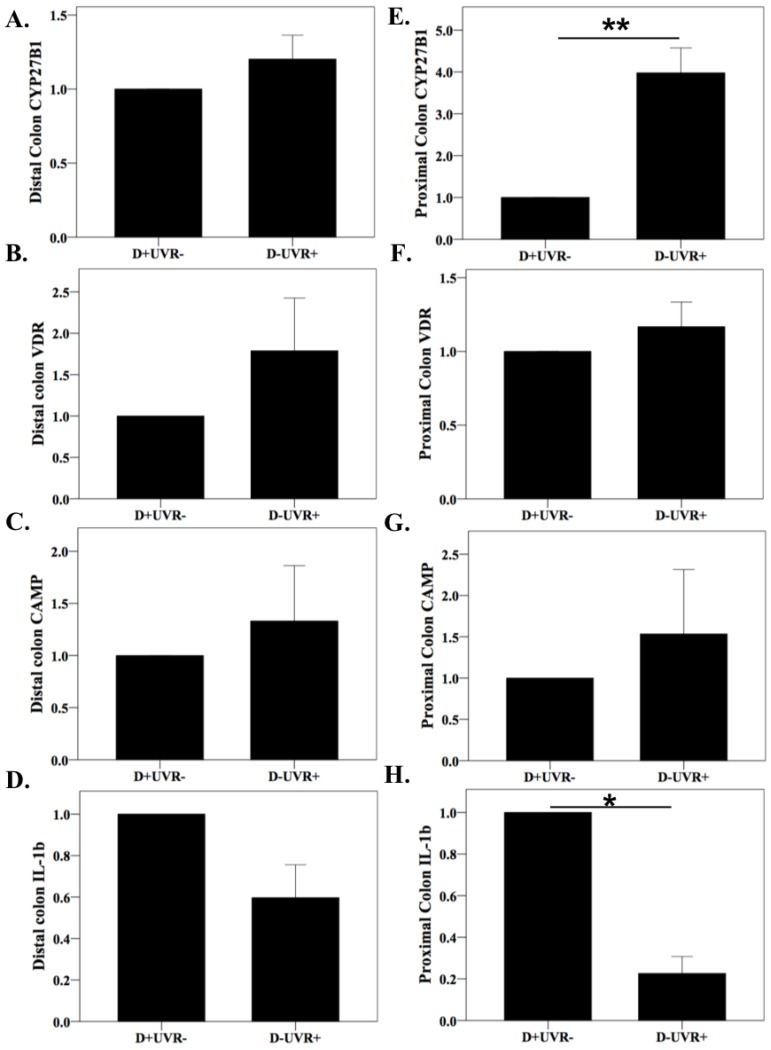
Colon *CYP27B1*, vitamin D receptor, cathelcidin, and *IL-1β* gene expression. At day 35, mice were sacrificed and colonic tissue harvested to determine gene expression in the distal (**A–D**) and proximal (**E–H**) colon from mice acquiring vitamin D exclusively through diet (D+UVR−) or UV irradiation (D−UVR+). Data are expressed as fold-change with the D+UVR− group as the control. mRNA gene expression by qPCR was calculated using the 2^−ΔΔCT^ method with TATA-box-binding protein as the housekeeping gene, *n =* 3/group. Values are expressed as mean ± SEM. * *p* < 0.05, ** *p* < 0.01.

Given the change in microbiota with UVR treatment, changes were sought in the gene expression of colonic *CYP27B1*, vitamin D receptor (*VDR*), cathelcidin antimicrobial peptide (*CAMP*), and *IL-1β*. Specifically, colons from mice from the D+UVR- and D-UVR+ groups were examined as these groups had similar circulating 25(OH)D_3_ levels, but vitamin D was acquired either exclusively from diet or UVR exposure. CYP27B1 is the 1-alpha hydroxylase responsible for the local activation of 25(OH)D to 1,25(OH)_2_D_3_. Expression of *CYP27B1* was four-fold greater among UV-irradiated mice compared to nonirradiated mice in the proximal but not distal colon (*p* = 0.007) (Figure 3A,E). Vitamin D receptor gene expression did not differ between groups (Figure 3B,F). As 1,25(OH)_2_D_3_ induces the cathelcidin gene to produce antimicrobial peptides (CAMP), the gene expression of *CAMP* was examined and found to be similar among mice from the UVR and non-UVR groups (Figure 3C,G). The mRNA expression of *IL-1β*, encoding the proinflammatory cytokine IL-1β, was reduced almost five-fold among UV-irradiated compared to the nonirradiated mice in the proximal (*p* = 0.011) but not distal colon (Figure 3D,H). Gene expression of *CYP27B1* in kidney tissue was 1.8-fold greater among D−UVR+ compared to D+UVR− mice, but this was not statistically significant (*p* = 0.2), and serum 1,25(OH)_2_D_3_ levels were similar between groups (Supplementary Figure S3).

### 3.4. The Effect of UV Irradiation of Skin on DSS Colitis

After six days of DSS treatment (days 28 to 34), all mice underwent colonoscopy (*n* = 35/group) and were sacrificed at days 35, 42, 49, and 63 (Supplementary Figure S1). As expected, all DSS-treated mice lost weight and liquid stools were observed. We previously reported worse colitis in D++ compared to D+ mice as measured by colonoscopy, weight loss, and histology, and there was no improvement in colitis severity among D− mice exposed to UVR [7].

**Figure 4:**
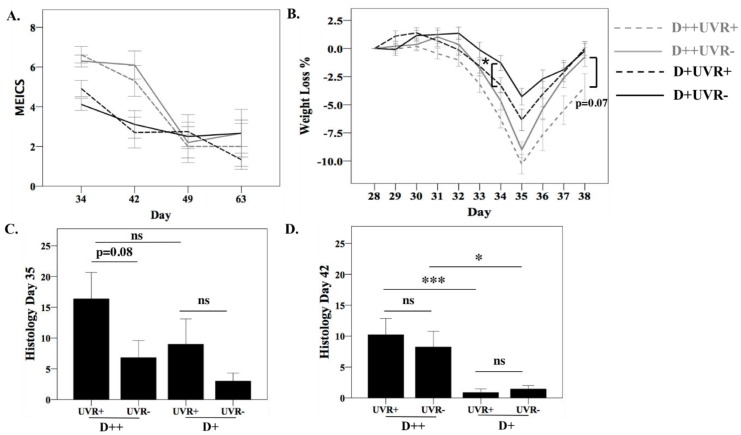
Effect of UV irradiation of skin on dextran sodium sulfate (DSS) colitis. After mice were established on respective diets for 28 days and half of each group received 4 daily doses of 1 kJ/m^2^ UVR, mice were treated orally with DSS 3% for 6 days. Colitis was measured by (**A**) colonoscopy calculating the murine endoscopic index of severity (MEICS), with *n* = 35/group for day 34, *n* = 10/group for day 42, and *n* = 5/group for assessments at days 49 and 63; (**B**) percentage weight loss; *p*-value comparison was for UVR vs no UVR within the same vitamin D diet group; and (**C**) histological severity score at day 35 and (**D**) day 42, *n* = 7–10/group. Values are shown as mean ± SEM. * *p* < 0.05, *** *p* < 0.001.

In the current study, D++ mice treated with or without UVR demonstrated significantly greater endoscopic evidence of colitis (MEICS) and greater weight loss at days 34 and 35, compared to their D+ counterparts (Figure 4A,B). Among the D++ and D+ groups, UV irradiation of the mice did not alter the initial severity of colitis or the speed of recovery over time compared to mice without UVR exposure, with similar MEICS scores at all timepoints. At day 34, greater weight loss was recorded among D+UVR+ mice compared to D+UVR− mice (*p* = 0.04). At day 38, there was a trend for D++UVR+ mice to have regained less weight compared to D++UVR− with persisting 5.3 ± 1.7% versus 1.2 ± 1.4% weight loss compared to baseline (*p* = 0.07).

Histological colitis was more severe among D++ mice compared to D+ mice irrespective of UVR exposure; these changes were statistically significant at day 42 (Figure 4C,D). At day 35, there was a trend for worse colitis among UVR-irradiated D++ mice compared to D++ UV nonirradiated mice (*p* = 0.08). At day 42, there were no differences detected between UV-irradiated and nonirradiated groups.

### 3.5. Microbiome in Faecal Samples from DSS Mice

The effect of UV irradiation on the faecal microbiome from all groups treated with DSS was explored. Faecal samples from 28 mice were collected at day 35 (seven days after DSS initiation). These comprised five samples from each of the six groups, except D++UVR+ and D+UVR+, where four samples were collected. No differences were seen in alpha-diversity between groups.

The overall beta-diversity was examined by PERMANOVA (Table 3). Significant differences were detected with UV irradiation of vitamin D-deficient mice (D−UVR+ vs D−UVR−, *t* = 1.42, *p* = 0.034). Furthermore, when comparing mice that exclusively derived vitamin D through UVR exposure (D−UVR+) to mice receiving only dietary vitamin D (D+UVR−), a significant difference was seen (*t* = 1.52, *p* = 0.021) despite the mice having similar serum 25(OH)D and 1,25(OH)_2_D_3_ levels (Supplementary Table S1 and Supplementary Figure S3).

**Figure 5:**
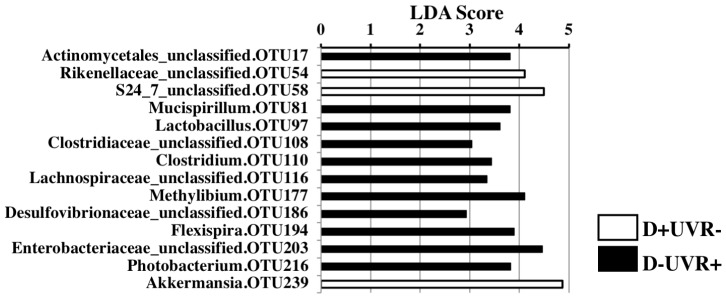
The effect of UV irradiation of skin versus dietary vitamin D on microbial composition of faecal samples post-DSS. Linear discrimination analysis effect size (LDA score) was determined for evaluation of significant differences in the relative abundance of individual taxa in faeces from DSS mice (day 35) that acquired vitamin D following skin exposure to UVR (D−UVR+) versus those mice acquiring vitamin D through diet alone (D+UVR−). Only taxa where a significant change (*p* < 0.05) was observed are illustrated; *n* = 5/group.

Changes in individual taxa were also examined in faecal pellets from the DSS mice. In vitamin D-deficient mice exposed to UVR (D−UVR+), an overall shift from phylum Bacteroidetes to phyla Firmicutes and Verrucomicrobia was detected in faecal pellets compared to those from nonirradiated counterparts (D−UVR−) at day 35 using linear discriminant analysis (LEfSe) (Supplementary Table S1). Comparing the faecal microbiome of mice deriving vitamin D from UVR (D−UVR+) compared to diet (D+UVR−), there were a number of taxa reaching LDA scores greater than 3.5, including Actinomycetales (*p* = 0.005), *Mucispirillum* (*p* = 0.047), *Lactobacillus* (*p* = 0.009), *Methylibium* (*p* = 0.005), *Flexispira* (*p* = 0.028), *Enterobacteraceae* (*p* = 0.047), and *Photobacterium* (*p* = 0.019) (Figure 5). Conversely, the faecal microbiome of mice in the D+UVR− group had greater relative abundance of *Rickenellaceae* (*p* = 0.005), S24_7 (*p* = 0.047), and *Akkermansia* (*p* = 0.028), which all had LDA scores >4.

## 4. Discussion

Dietary interventions including oral vitamin D supplementation can have significant effects on the intestinal microbiome [25]. To our knowledge, this is the first study to explore the effect of UVR on the faecal microbiome. When the effect of UV irradiation within each vitamin D group was examined, there were no significant changes in alpha- or beta-diversity measures, but this is likely due to the study being underpowered to detect this effect. When the faecal microbiomes of UV-irradiated mice were collectively examined, significant differences were observed compared to those of nonirradiated mice, even after controlling for vitamin D group and time of sacrifice. At the phylum level, there was a shift from Bacteroidetes to Deferribacteres and Firmicutes. The genus *Mucispirillum* was enriched with UVR exposure (*p* = 0.04): *Mucispirillia* are abundant inhabitants of the intestinal mucus layer of rodents and other animals which possess mucolytic activity and are increased during inflammation [26,27]. A loss of mucus secretion due to *Mucispirillia* may increase sensitivity to chemical adjuvants, and in some cases, induce spontaneous colitis [28,29,30]. Among Firmicutes, the genus *Coprococcus* was enriched with UVR exposure. This bacterium produces butyric acid, a short-chain fatty acid with known anti-inflammatory effects which is depleted in paediatric patients with active inflammatory bowel disease [31]. Interestingly, we recently reported the depletion of the genus *Coprococcus* with high vitamin D diets, which was also associated with more severe DSS colitis [7], again suggesting different effects of UVR versus dietary vitamin D on specific bacterial taxa. Members of the genus *Clostridium* were also enriched in the faecal samples from UV-irradiated mice; these species are commensals within the gastrointestinal tract, however they include well-recognised pathogens such as *Clostridium difficile*. Among the phylum Proteobacteria, there was a shift from Alphaproteobacteria to Deltaproteobacteria, with the genus *Desulfovibrio* in particular well represented with UVR exposure. *Desulfovibrio* spp. have the capacity to metabolise colonic mucin. These bacteria may contribute to mucosal inflammation in UC through production of potentially toxic hydrogen sulfide, released as a by-product of the metabolism of sulfated mucin [32]. As is evident here, there were mixed changes in the faecal microbiome and according to current knowledge, some may be beneficial and others detrimental.

Vitamin D-sufficient (D+) mice exposed to UVR had a lower level of serum IL-17 compared to nonirradiated mice, with a trend to statistical significance (*p* = 0.05). This effect of UVR was also noted among mice on high vitamin D diets (D++), although this was again not statistically significant, likely due to the high level of variability. This is consistent with the current understanding that UVR can inhibit T-cell proliferation and suppress antigen-specific responses involving Th1, Th17, and Th2 cells [33,34]. IL-17 is increased in human inflammatory bowel disease [35]. IL-17A knockout (KO) mice were protected against 2,4,6-Trinitrobenzenesulfonic acid solution (TNBS)-induced colitis, and similarly, IL-17F KO mice had less severe DSS-colitis; however, interestingly, IL-17A KO mice had worse DSS-induced inflammation [36]. Furthermore, a clinical trial of the anti-IL-17 monoclonal antibody secukinumab led to worse outcomes in Crohn’s disease [37]. As a result, no further trials were carried out for this drug in IBD, but it remains a proven treatment option in other autoimmune conditions such as psoriasis [37,38].

Mice deriving vitamin D through exposure to UVR (D−UVR+) had greater *CYP27B1* gene expression in the proximal colon than was measured in vitamin D-sufficient mice not exposed to UVR (D+UVR−). This suggested that UVR may have stimulated increased 1,25(OH)_2_D_3_ synthesis in the colon and may explain local effects on luminal microbiota. To our knowledge, prior studies have not examined colonic gene expression of *CYP27B1* after UV irradiation of shaved skin. In a previous study, greater *CYP27B1* expression was measured in the distal rather than the proximal colon, but treatment with DSS induced greater expression of the enzyme in the proximal rather than the distal colon [39]. While not specific to UVR exposure, these previous findings support the concept of differential *CYP27B1* expression in the proximal compared to the distal colon with different environmental exposures. Increased *CYP27B1* expression is expected to cause higher 1,25(OH)_2_D_3_ levels and activation of downstream pathways to promote the intracellular killing of bacteria, attenuation of dendritic cell capacity for antigen presentation, promotion of IL-10, and inhibition of IL-12 expression [40]. Recent data support a fundamental role for IL-1β in Th17 modulation, with IL-1β able to induce the expression of transcription factors necessary for Th17 development [41]. Thus, the reduced proximal colon expression of *IL-1β* in UV-irradiated mice is consistent with the lower levels of circulating IL-17 also measured in the UV-irradiated mice.

UVR exposure did not protect against DSS colitis. There was greater weight loss in the D+UVR+ versus D+UVR− group at day 34, but not at other timepoints, and there was a nonsignificant increase in histological colitis severity in the D++UVR+ versus D++UVR− group. In contrast, results from two other studies of light therapy in DSS colitis showed a protective effect. In the first, unshaven mice were treated either with no phototherapy, low light (1000 lux), or high light (2500 lux) phototherapy for one week starting on the second day of DSS colitis [42]. Phototherapy was delivered for 12 h/day, but the wavelengths used were not described. They found reduced colitis severity in the low but not the high light group, though 1,25(OH)_2_D was only increased in the high light group [42]. The second study irradiated C57BL/6 shaven mice (eight/group) with 1.5 kJ/m^2^ broadband UVR (280–350 nm) daily for four days prior to treatment with 2.5% DSS for eight days [43]. There was significantly less weight loss, reduced faecal hemoccult blood, and histological colitis scores in UV-irradiated mice. The reason for the conflicting results is not clear; there were differences in the measurement of colitis severity and the doses of UVR given (1.5 kJ/m^2^ as opposed to 1 kJ/m^2^ in our study). The strength of our results is that greater numbers of mice were examined and that colitis was assessed objectively with blinded endoscopic and histologic measurement. Overall, the DSS model of colitis may not be the ideal experimental model to determine the benefits of UVR exposure. DSS induces chemical trauma to the colonic mucosa to generate colitis, and thus is a better model for studying innate immune responses in IBD. UVR, on the other hand, has effects on both innate and adaptive immune responses, but its effect on T cells and their involvement in the adaptive immune system plays a major role in its ability to suppress systemic immunity. Thus, models such as the IL-10 KO and *TNF^ΔΔRE^,* which develop intestinal inflammation due to defects in adaptive immunity, may be better suited to exploring the benefits of skin exposure to UVR [10].

The impact of vitamin D on DSS colitis and the faecal microbiome has been previously reported by us [7] and others [44,45], and recently also in a small series of IBD patients [46,47]. In the current study, UVR was associated with changes in the faecal microbial composition after DSS exposure that differed from the changes described in non-DSS mice. Notably, there was a significant change in beta-diversity between D−UV+ compared to the D+UV− group despite similar serum 25(OH)D levels. This difference could be attributed to the vitamin D-independent effects of UVR, or alternatively, it may reflect the mode of acquiring vitamin D. Dietary vitamin D, by virtue of direct contact with the intestinal mucosa and microbiota, may have a different impact on the intestinal microbial composition as opposed to vitamin D acquired via UVR/dermal synthesis and reaching the intestinal mucosa through the systemic circulation. Interpreting the wide range of changes in specific taxa after DSS treatment is challenging, but notably in mice acquiring vitamin D only through diet (D+UVR−) compared to UVR (D−UVR+), there was enrichment of *Verrucomicroiaceae*, *Akkermansia*, and the recently described Bacteroidales S24-7. A recent study comparing the effects of *Salmonella* infection and DSS colitis demonstrated enrichment of these taxa in mice with low-level inflammation secondary to *Salmonella* infection or DSS [48]. *Akkermansia* are known to degrade mucin as their sole carbon and nitrogen source, and these bacteria increased after low levels of inflammation, when mucin may be produced. S24-7 have similar mucin degradation capacity. Both *Akkermansia* and members of family S24-7 encode the capacity for propionate production, which may stabilise inflammation in the gut, forming a positive feedback loop [49]. Thus, while induced by inflammation, their metabolic activity may act to limit colitis. Conversely, the D−UVR+ faecal microbiome was enriched with members of Clostridia, Helicobacter, and Enterobacteriaceae, which may potentiate inflammation, as well as Lactobacilli, which are often used as a probiotic. The ‘net effect’ of these microbial changes on inflammation is difficult to determine, but these data suggest that when faced with an insult such as DSS colitis, there are changes in the faecal microbiome of UV-irradiated mice that are different to those measured in mice acquiring vitamin D exclusively through dietary sources.

Some study limitations are noteworthy. There was a relatively limited sample size per group examined for faecal microbiota analysis at each timepoint (*n* = 5/group); this was due to the large number of study groups—12 in total—necessitating fewer samples per group for feasibility. A sample size of five per group, however, is in keeping with previously published and frequently cited murine microbiome studies [28]. Notwithstanding this, in the absence of colitis, the sample size limited the ability to detect changes in faecal microbial composition from mice on individual diets +/− UVR. As described, however, when the groups were analysed together (*n* = 60), clear differences in microbial composition were seen with UV-irradiation and controlling for vitamin D groups. Furthermore, as the study was only conducted in female mice, the study findings may not be generalisable to male mice. We acknowledge the impact gender may have on vitamin D metabolism as described in both human and animal studies, and future studies should aim to replicate these findings in both male and female mice [11,50].

In conclusion, both diet and UVR (as in sunlight) were effective methods of acquiring vitamin D; however, they were associated with different effects on the faecal microbiome in the healthy state and after an insult such as DSS colitis. These differential effects may have significance in health and disease, including inflammatory bowel disease, and further experimentation with alternative animal models are warranted.

## Figures and Tables

**Table 1:** Effect of UV irradiation of skin on overall beta-diversity in faecal samples.

Day/Treatment	Group 1	Group 2	*t*-Value	*p-*Value
**Day 35**	D++ UVR+	D++ UVR−	1.24	0.15
	D+ UVR+	D+ UVR−	0.79	0.84
	D− UVR+	D− UVR−	0.89	0.67
	D− UVR+	D+ UVR−	0.86	0.67
**Day 63**	D++ UVR+	D++ UVR−	1.09	0.30
	D+ UVR+	D+ UVR−	1.03	0.39
	D− UVR+	D− UVR−	0.79	0.84
	D− UVR+	D+ UVR−	0.82	0.79

PERMANOVA was used to calculate the effect of UVR on overall beta-diversity in faecal samples from mice within each vitamin D group. A separate analysis of mice acquiring vitamin D solely through UVR (D−UVR+) versus diet (D+UVR−) is shown in the fourth line of each group. *n* = 5/group, degrees of freedom (df) = 7–8 in all analyses, and permutations = 126. “*t*” = *t*-statistic.

**Table 2:** Serum cytokines and skin exposure to UV irradiation.

	D++UVR+	D++UVR−	*p*	D+UVR+	D+UVR−	*p*	D−UVR+	D−UVR−	*p*
IL-1β (pg/mL)	35.0 ± 70.0	97.3 ± 194.6	ns	0	226.0 ± 226.0	ns	0	86.9 ± 78.0	ns
TNF-α (pg/mL)	335.4 ± 120.6	411.3 ± 274.2	ns	301.6 ± 99.5	392.2 ± 161.2	ns	230.2 ± 19.1	356.4 ± 107.6	ns
IL-10 (pg/mL)	53.8 ± 20.1	66.9 ± 31.9	ns	51.9 ± 3.8	98.1 ± 60.8	ns	40.4 ± 3.3	57.2 ± 12.2	ns
IL-6 (pg/mL)	7.9 ± 3.6	7.8 ± 7.2	ns	6.7 ± 1.0	18.5 ± 14.5	ns	6.1 ± 1.6	7.2 ± 3.1	ns
IL-17 (pg/mL)	146.2 ± 38.5	177.6 ± 53.2	ns	116.7 ± 30.1	207.9 ± 72.7	0.05	121.5 ± 12.0	159.7 ± 24.8	ns

The effect of UVR of skin on serum IL-1β, TNF-α, IL-10, IL-6, and IL-17 was examined in mice on the D++, D+, and D− diets. Values are represented as mean ± SD. *n* = 3–4/group. ns = not significant.

**Table 3:** Effect of UV irradiation of skin on overall beta-diversity in faecal samples from DSS mice at day 35.

Group 1	Group 2	*t*	*p*-Value
D++ UVR+	D++ UVR−	0.92	0.61
D+ UVR+	D+ UVR−	1.17	0.26
D− UVR+	D− UVR−	1.42	0.034
D− UVR+	D+ UVR−	1.52	0.021

PERMANOVA was used to calculate the effect of UVR on overall beta-diversity in faecal samples from mice within each vitamin D group after treatment for 6 days with DSS (day 35). A separate analysis of mice acquiring vitamin D solely through UVR (D−UVR+) versus diet (D+UVR−) is shown in the fourth line. *n* = 4–5/group, degrees of freedom (df) = 7–8 in all analyses, and permutations = 126.

## Supplementary Materials

The following are available online at http://www.mdpi.com/2072-6643/10/8/1069/s1, Figure S1: The experimental approach, Figure S2: Serum 25(OH)D_3_ levels, Figure S3: Kidney *CYP27B1* gene expression and 1,25(OH)_2_D_3_ levels, Table S1: LEfSe analyses comparing mice acquiring vitamin D either exclusively through diet versus UV-irradiation.

## References

[1] Shivananda S., Lennard-Jones J., Logan R., Fear N., Price A., Carpenter L., Blankenstein M.V. (1996). Incidence of inflammatory bowel disease across europe: Is there a difference between north and south? Results of the european collaborative study on inflammatory bowel disease (EC–IBD). Gut.

[2] Gower-Rousseau C., Salomez J.L., Dupas J.L., Marti R., Nuttens M.C., Votte A., Lemahieu M., Lemaire B., Colombel J.F., Cortot A. (1994). Incidence of inflammatory bowel disease in northern france (1988–1990). Gut.

[3] Sonnenberg A. (2009). Demographic characteristics of hospitalized IBD patients. Dig. Dis. Sci..

[4] Nerich V., Monnet E., Etienne A., Louafi S., Ramée C., Rican S., Weill A., Vallier N., Vanbockstael V., Auleley G.-R. (2006). Geographical variations of inflammatory bowel disease in france: A study based on national health insurance data. Inflamm. Bowel Dis..

[5] Nerich V., Jantchou P., Boutron-Ruault M.-C., Monnet E., Weill A., Vanbockstael V., Auleley G.-R., Balaire C., Dubost P., Rican S. (2011). Low exposure to sunlight is a risk factor for Crohn’s disease. Aliment. Pharmacol. Ther..

[6] Assa A., Vong L., Pinnell L.J., Avitzur N., Johnson-Henry K.C., Sherman P.M. (2014). Vitamin d deficiency promotes epithelial barrier dysfunction and intestinal inflammation. J. Infect. Dis..

[7] Ghaly S., Kaakoush N.O., Lloyd F., McGonigle T., Mok D., Baird A., Klopcic B., Gordon L., Gorman S., Forest C. (2018). High dose vitamin D supplementation alters faecal microbiome and predisposes mice to more severe colitis. Sci. Rep..

[8] Hart P.H., Gorman S., Finlay-Jones J.J. (2011). Modulation of the immune system by UV radiation: More than just the effects of vitamin d?. Nat. Rev. Immunol..

[9] Ullrich S.E., Byrne S.N. (2012). The immunologic revolution: Photoimmunology. J. Investig. Dermatol..

[10] Wirtz S., Neurath M.F. (2007). Mouse models of inflammatory bowel disease. Adv. Drug Deliv. Rev..

[11] Gorman S., Scott N.M., Tan D.H., Weeden C.E., Tuckey R.C., Bisley J.L., Grimbaldeston M.A., Hart P.H. (2012). Acute erythemal ultraviolet radiation causes systemic immunosuppression in the absence of increased 25-hydroxyvitamin D3 levels in male mice. PLoS ONE.

[12] Geldenhuys S., Hart P.H., Endersby R., Jacoby P., Feelisch M., Weller R.B., Matthews V., Gorman S. (2014). Ultraviolet radiation suppresses obesity and symptoms of metabolic syndrome independently of vitamin D in mice fed a high-fat diet. Diabetes.

[13] Zhang J., Kobert K., Flouri T., Stamatakis A. (2014). Pear: A fast and accurate illumina paired-end read merger. Bioinformatics.

[14] Kim M., Jee S.H., Kim M., Yoo H.J., Kang M., Kim J., Lee J.H. (2017). Serum vitamin A-related metabolite levels are associated with incidence of type 2 diabetes. Dia. Metabol..

[15] Caporaso J.G., Kuczynski J., Stombaugh J., Bittinger K., Bushman F.D., Costello E.K., Fierer N., Pena A.G., Goodrich J.K., Gordon J.I. (2010). Qiime allows analysis of high-throughput community sequencing data. Nat. Methods.

[16] Edgar R.C. (2010). Search and clustering orders of magnitude faster than blast. Bioinformatics.

[17] Edgar R.C., Haas B.J., Clemente J.C., Quince C., Knight R. (2011). Uchime improves sensitivity and speed of chimera detection. Bioinformatics.

[18] DeSantis T.Z., Hugenholtz P., Larsen N., Rojas M., Brodie E.L., Keller K., Huber T., Dalevi D., Hu P., Andersen G.L. (2006). Greengenes, a chimera-checked 16s rRNA gene database and workbench compatible with ARB. Appl. Environ. Microbiol..

[19] Segata N., Izard J., Waldron L., Gevers D., Miropolsky L., Garrett W.S., Huttenhower C. (2011). Metagenomic biomarker discovery and explanation. Genome. Biol..

[20] Gorman S., Judge M.A., Hart P.H. (2010). Topical 1,25-dihydroxyvitamin D3 subverts the priming ability of draining lymph node dendritic cells. Immunology.

[21] Ng Y.-L., Klopcic B., Lloyd F., Forrest C., Greene W., Lawrance I.C. (2013). Secreted protein acidic and rich in cysteine (SPARC) exacerbates colonic inflammatory symptoms in dextran sodium sulphate-induced murine colitis. PLoS ONE.

[22] Becker C., Fantini M.C., Wirtz S., Nikolaev A., Kiesslich R., Lehr H.A., Galle P.R., Neurath M.F. (2005). In vivo imaging of colitis and colon cancer development in mice using high resolution chromoendoscopy. Gut.

[23] Dieleman L.A., Palmen M.J., Akol H., Bloemena E., Peña A.S., Meuwissen S.G., Van Rees E.P. (1998). Chronic experimental colitis induced by dextran sulphate sodium (DSS) is characterized by th1 and th2 cytokines. Clin. Exp. Immunol..

[24] Clarke M.W., Tuckey R.C., Gorman S., Holt B., Hart P.H. (2013). Optimized 25-hydroxyvitamin d analysis using liquid-liquid extraction with 2D separation with LC/MS/MS detection, provides superior precision compared to conventional assays. Metabolomics.

[25] Bashir M., Prietl B., Tauschmann M., Mautner S.I., Kump P.K., Treiber G., Wurm P., Gorkiewicz G., Högenauer C., Pieber T.R. (2015). Effects of high doses of vitamin D3 on mucosa-associated gut microbiome vary between regions of the human gastrointestinal tract. Eur. J. Nutr..

[26] Png C.W., Linden S.K., Gilshenan K.S., Zoetendal E.G., McSweeney C.S., Sly L.I., McGuckin M.A., Florin T.H. (2010). Mucolytic bacteria with increased prevalence in IBD mucosa augment in vitro utilization of mucin by other bacteria. Am. J. Gastroenterol..

[27] Loy A., Pfann C., Steinberger M., Hanson B., Herp S., Brugiroux S., Gomes Neto J.C., Boekschoten M.V., Schwab C., Urich T. (2017). Lifestyle and horizontal gene transfer-mediated evolution of mucispirillum schaedleri, a core member of the murine gut microbiota. Msystems.

[28] Berry D., Schwab C., Milinovich G., Reichert J., Ben Mahfoudh K., Decker T., Engel M., Hai B., Hainzl E., Heider S. (2012). Phylotype-level 16s rRNA analysis reveals new bacterial indicators of health state in acute murine colitis. ISME J..

[29] Van der Sluis M., De Koning B.A., De Bruijn A.C., Velcich A., Meijerink J.P., Van Goudoever J.B., Buller H.A., Dekker J., Van Seuningen I., Renes I.B. (2006). Muc2-deficient mice spontaneously develop colitis, indicating that MUC2 is critical for colonic protection. Gastroenterology.

[30] Heazlewood C.K., Cook M.C., Eri R., Price G.R., Tauro S.B., Taupin D., Thornton D.J., Png C.W., Crockford T.L., Cornall R.J. (2008). Aberrant mucin assembly in mice causes endoplasmic reticulum stress and spontaneous inflammation resembling ulcerative colitis. PLoS Med..

[31] Shaw K.A., Bertha M., Hofmekler T., Chopra P., Vatanen T., Srivatsa A., Prince J., Kumar A., Sauer C., Zwick M.E. (2016). Dysbiosis, inflammation, and response to treatment: A longitudinal study of pediatric subjects with newly diagnosed inflammatory bowel disease. Genome Med..

[32] Earley H., Lennon G., Balfe A., Kilcoyne M., Clyne M., Joshi L., Carrington S., Martin S.T., Coffey J.C., Winter D.C. (2015). A preliminary study examining the binding capacity of akkermansia muciniphila and desulfovibrio spp., to colonic mucin in health and ulcerative colitis. PLoS ONE.

[33] Schwarz T. (2005). Mechanisms of UV–induced immunosuppression. Keio J. Med..

[34] Gorman S., McGlade J.P., Lambert M.J., Strickland D.H., Thomas J.A., Hart P.H. (2010). UV exposure and protection against allergic airways disease. Photochem. Photobiol. Sci..

[35] Catana C.S., Berindan Neagoe I., Cozma V., Magdas C., Tabaran F., Dumitrascu D.L. (2015). Contribution of the IL-17/IL-23 axis to the pathogenesis of inflammatory bowel disease. World J. Gastroenterol..

[36] Wedebye Schmidt E.G., Larsen H.L., Kristensen N.N., Poulsen S.S., Lynge Pedersen A.M., Claesson M.H., Pedersen A.E. (2013). Th17 cell induction and effects of IL-17a and IL-17f blockade in experimental colitis. Inflamm. Bowel Dis..

[37] Hueber W., Sands B.E., Lewitzky S., Vandemeulebroecke M., Reinisch W., Higgins P.D., Wehkamp J., Feagan B.G., Yao M.D., Karczewski M. (2012). Secukinumab, a human anti-IL-17a monoclonal antibody, for moderate to severe crohn’s disease: Unexpected results of a randomised, double-blind placebo-controlled trial. Gut.

[38] Bissonnette R., Luger T., Thaci D., Toth D., Lacombe A., Xia S., Mazur R., Patekar M., Charef P., Milutinovic M. (2018). Secukinumab demonstrates high sustained efficacy and a favorable safety profile in patients with moderate to severe psoriasis through 5 years of treatment (sculpture extension study). J. Eur. Acad. Dermatol. Venereol..

[39] Liu N., Nguyen L., Chun R.F., Lagishetty V., Ren S., Wu S., Hollis B., DeLuca H.F., Adams J.S., Hewison M. (2008). Altered endocrine and autocrine metabolism of vitamin D in a mouse model of gastrointestinal inflammation. Endocrinology.

[40] Ghaly S., Lawrance I. (2014). The role of vitamin D in gastrointestinal inflammation. Expert Rev. Gastroenterol. Hepatol..

[41] Santarlasci V., Cosmi L., Maggi L., Liotta F., Annunziato F. (2013). IL-1 and T helper immune responses. Front. Immunol..

[42] Hiratsuka T., Inomata M., Goto S., Oyama Y., Nakano T., Chen C.-L., Shiraishi N., Noguchi T., Kitano S. (2014). Phototherapy with artificial light suppresses dextran sulfate sodium–induced colitis in a mouse model. J. Gastroenterol. Hepatol..

[43] Breuer J., Schwab N., Schneider-Hohendorf T., Marziniak M., Mohan H., Bhatia U., Gross C.C., Clausen B.E., Weishaupt C., Luger T.A. (2014). Ultraviolet B light attenuates the systemic immune response in central nervous system autoimmunity. Ann. Neurol..

[44] Lagishetty V., Misharin A.V., Liu N.Q., Lisse T.S., Chun R.F., Ouyang Y., McLachlan S.M., Adams J.S., Hewison M. (2010). Vitamin D deficiency in mice impairs colonic antibacterial activity and predisposes to colitis. Endocrinology.

[45] Ooi J.H., Li Y., Rogers C.J., Cantorna M.T. (2013). Vitamin D regulates the gut microbiome and protects mice from dextran sodium sulfate-induced colitis. J. Nutr..

[46] Schaffler H., Herlemann D.P., Klinitzke P., Berlin P., Kreikemeyer B., Jaster R., Lamprecht G. (2018). Vitamin D administration leads to a shift of the intestinal bacterial composition in Crohn’s disease patients, but not in healthy controls. J. Dig. Dis..

[47] Garg M., Hendy P., Ding J.N., Shaw S., Hold G., Hart A. (2018). The effect of vitamin D on intestinal inflammation and faecal microbiota in patients with ulcerative colitis. J. Crohns Colitis.

[48] Borton M.A., Sabag-Daigle A., Wu J., Solden L.M., O’Banion B.S., Daly R.A., Wolfe R.A., Gonzalez J.F., Wysocki V.H., Ahmer B.M.M. (2017). Chemical and pathogen-induced inflammation disrupt the murine intestinal microbiome. Microbiome.

[49] Vinolo M.A., Rodrigues H.G., Nachbar R.T., Curi R. (2011). Regulation of inflammation by short chain fatty acids. Nutrients.

[50] Sharifi N., Amani R., Hajiani E., Cheraghian B. (2016). Women may respond different from men to vitamin D supplementation regarding cardiometabolic biomarkers. Exp. Biol. Med. (Maywood).

