# Supplementary material for: Ultraviolet Irradiation of Skin Alters the Faecal Microbiome Independently of Vitamin D in Mice
Source: Nutrients. 2018 Aug 11;10(8):1069. doi: 10.3390/nu10081069 (PMC6116187; doi:10.3390/nu10081069)
Supplement: Supplementary file 1 [file nutrients-10-01069-s001.pdf]

nutrients

MDPI

Supplementary Materials: Ultraviolet Irradiation of Skin Alters the Faecal Microbiome Independently of Vitamin D in Mice

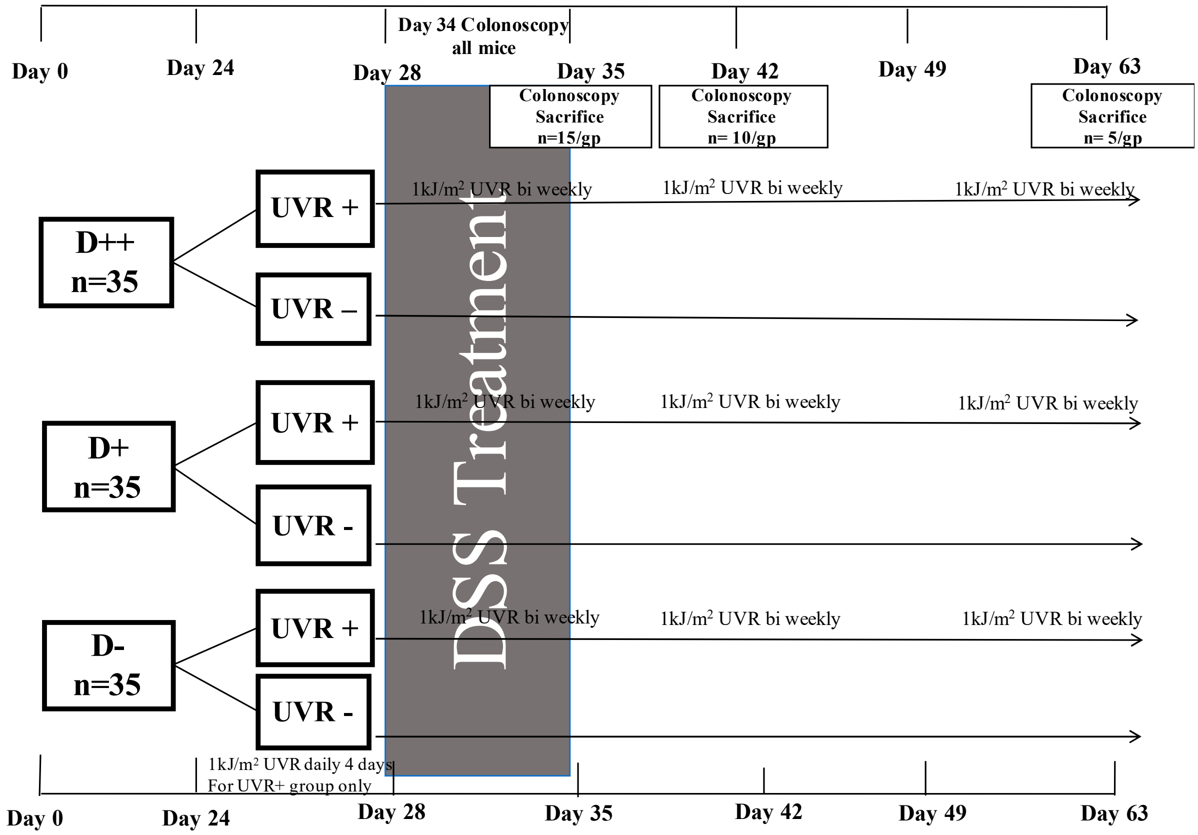

Figure S1. The experimental approach. C57BL/6 female mice (6-weeks-old) were fed diets with high (D++), moderate (D+) or no (D−) vitamin D3. After 24 days, half of the mice in each group were irradiated with 1kJ/m² UVR daily for 4 days, and then were maintained with twice weekly exposure to UVR for the remainder of the study. In the treatment group, after 28 days of dietary supplements, with or without UVR exposure (designated day 0), mice were given DSS orally for 6 days to induce colitis, and then were maintained without DSS and continued their respective vitamin D diet until day 63. All mice underwent colonoscopy at day 34, and were sacrificed at days 35, 42, 49 or 63. An additional colonoscopy was performed on days 42, 49 or 63 when mice were sacrificed.

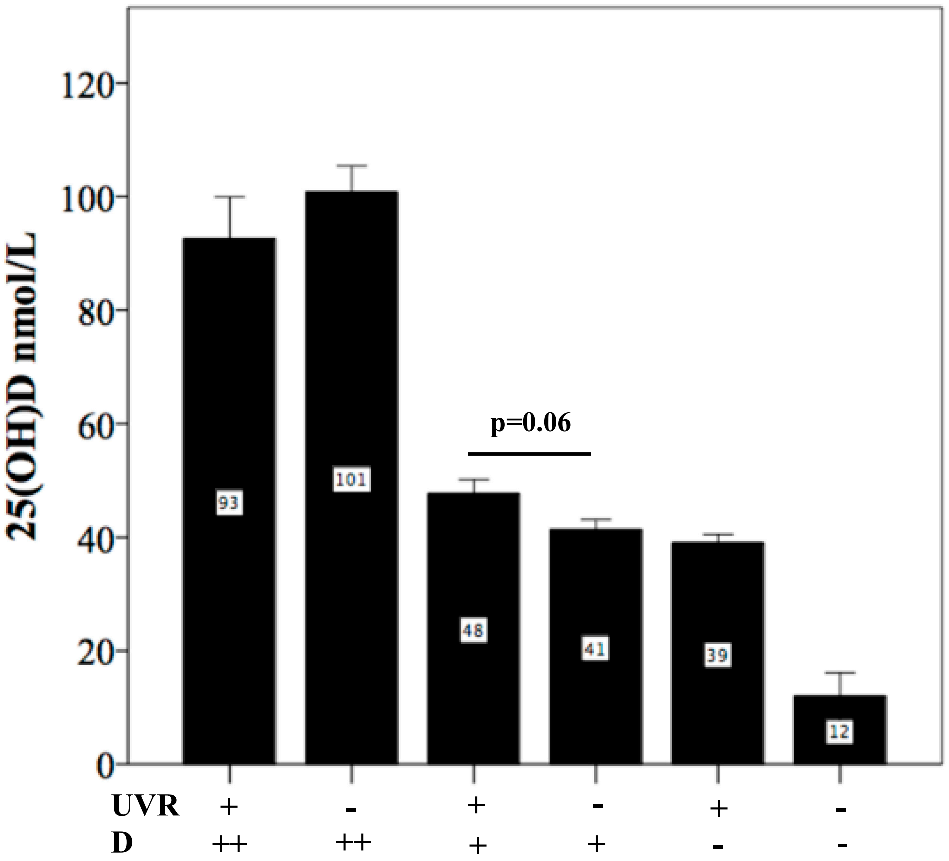

Figure S2. Serum 25(OH)D3 levels. Serum concentrations of 25(OH)D3 after 5 weeks on vitamin D diets and completing a total of 6 exposures to UVR (protocol day 7), n = 7–8/group. Values are expressed as mean ± SEM, from at least two experiments with the values shown in the bars. * $p < 0.05$, *** $p < 0.001$.

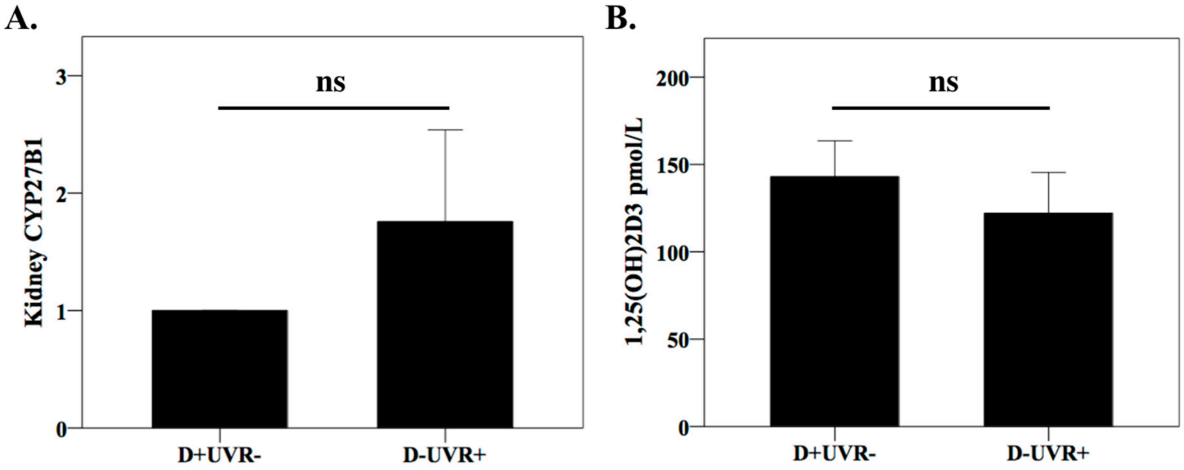

Figure S3. Kidney *CYP27B1* gene expression and 1,25(OH)2D3 levels. (**A**) Kidney tissue was harvested at day 35 to determine gene expression of CYP27B1. Data expressed as fold-change with the D+UVR− group as control. mRNA gene expression by qtPCR was calculated using the 2-ΔΔCT method with elongation factor 1 (eef1) as housekeeping gene. $n = 3$/group. (**B**) Serum 1,25(OH)2D3 measured at day 35, $n = 5$/group. Values are expressed as mean ± SEM, ns = not significant..

Supplementary Table S1. LEfSe analyses comparing mice acquiring vitamin D either exclusively through diet versus UV-irradiation.

Taxa	Group	LDA Score	*P*-Value
**Bacteria.Actinobacteria.Actinobacteriia.Actinomycetales**	D-UVR+	4.06	0.047
**Bacteria.Actinobacteria.Actinobacteriia.Actinomycetales.Actinomycetales_unclassified**	D-UVR+	3.98	0.013
**Bacteria.Actinobacteria.Actinobacteriia.Actinomycetales.Actinomycetales_unclassified.Actinomycetales_unclassified**	D-UVR+	3.97	0.013
**Bacteria.Actinobacteria.Actinobacteriia.Actinomycetales.Actinomycetales_unclassified.Actinomycetales_unclassified.OTU17**	D-UVR+	3.82	0.005
**Bacteria.Deferribacteres**	D-UVR+	3.82	0.047
**Bacteria.Deferribacteres.Deferribacteres**	D-UVR+	3.82	0.047
**Bacteria.Deferribacteres.Deferribacteres.Deferribacterales**	D-UVR+	3.82	0.047
**Bacteria.Deferribacteres.Deferribacteres.Deferribacterales.Deferribacteraceae**	D-UVR+	3.82	0.047
**Bacteria.Deferribacteres.Deferribacteres.Deferribacterales.Deferribacteraceae.Mucispirillum**	D-UVR+	3.82	0.047
**Bacteria.Deferribacteres.Deferribacteres.Deferribacterales.Deferribacteraceae.Mucispirillum.OTU81**	D-UVR+	3.82	0.047
**Bacteria.Firmicutes.Bacilli**	D-UVR+	3.82	0.028
**Bacteria.Firmicutes.Bacilli.Lactobacillales**	D-UVR+	3.61	0.009
**Bacteria.Firmicutes.Bacilli.Lactobacillales.Lactobacillaceae**	D-UVR+	3.60	0.009
**Bacteria.Firmicutes.Bacilli.Lactobacillales.Lactobacillaceae.Lactobacillus**	D-UVR+	3.60	0.009
**Bacteria.Firmicutes.Bacilli.Lactobacillales.Lactobacillaceae.Lactobacillus.OTU97**	D-UVR+	3.61	0.009
**Bacteria.Firmicutes.Clostridia.Clostridiales.Clostridiaceae.Clostridiaceae_unclassified.OTU108**	D-UVR+	3.05	0.007
**Bacteria.Firmicutes.Clostridia.Clostridiales.Clostridiaceae.Clostridium**	D-UVR+	3.44	0.007
**Bacteria.Firmicutes.Clostridia.Clostridiales.Clostridiaceae.Clostridium.OTU110**	D-UVR+	3.44	0.007
**Bacteria.Firmicutes.Clostridia.Clostridiales.Clostridiales_unclassified**	D-UVR+	4.58	0.047
**Bacteria.Firmicutes.Clostridia.Clostridiales.Clostridiales_unclassified.Clostridiales_unclassified**	D-UVR+	4.58	0.047
**Bacteria.Firmicutes.Clostridia.Clostridiales.Lachnospiraceae.Lachnospiraceae_unclassified.OTU116**	D-UVR+	3.35	0.016
**Bacteria.Proteobacteria**	D-UVR+	4.72	0.016
**Bacteria.Proteobacteria.Betaproteobacteria.Burkholderiales.Comamonadaceae**	D-UVR+	3.77	0.005
**Bacteria.Proteobacteria.Betaproteobacteria.Burkholderiales.Comamonadaceae.Methylibium**	D-UVR+	4.12	0.005
**Bacteria.Proteobacteria.Betaproteobacteria.Burkholderiales.Comamonadaceae.Methylibium.OTU177**	D-UVR+	4.11	0.005
**Bacteria.Proteobacteria.Deltaproteobacteria.Desulfovibrionales.Desulfovibrionaceae.Desulfovibrionaceae_unclassified**	D-UVR+	2.91	0.0283

**Bacteria.Proteobacteria.Deltaproteobacteria.Desulfovibrionales.Desulfovibrionaceae.Desulfovibrionaceae_unclassified.OTU186**	D-UVR+	2.93	0.0283
**Bacteria.Proteobacteria.Epsilonproteobacteria**	D-UVR+	3.90	0.0283
**Bacteria.Proteobacteria.Epsilonproteobacteria.Campylobacterales**	D-UVR+	3.90	0.0283
**Bacteria.Proteobacteria.Epsilonproteobacteria.Campylobacterales.Helicobacteraceae**	D-UVR+	3.90	0.0283
**Bacteria.Proteobacteria.Epsilonproteobacteria.Campylobacterales.Helicobacteraceae.Flexispira**	D-UVR+	3.90	0.0283
**Bacteria.Proteobacteria.Epsilonproteobacteria.Campylobacterales.Helicobacteraceae.Flexispira.OTU194**	D-UVR+	3.90	0.0283
**Bacteria.Proteobacteria.Gammaproteobacteria**	D-UVR+	4.46	0.0472
**Bacteria.Proteobacteria.Gammaproteobacteria.Enterobacteriales**	D-UVR+	4.46	0.0472
**Bacteria.Proteobacteria.Gammaproteobacteria.Enterobacteriales.Enterobacteriaceae**	D-UVR+	4.46	0.0472
**Bacteria.Proteobacteria.Gammaproteobacteria.Enterobacteriales.Enterobacteriaceae.Enterobacteriaceae_unclassified**	D-UVR+	4.46	0.0472
**Bacteria.Proteobacteria.Gammaproteobacteria.Enterobacteriales.Enterobacteriaceae.Enterobacteriaceae_unclassified.OTU203**	D-UVR+	4.46	0.0472
**Bacteria.Proteobacteria.Gammaproteobacteria.Vibrionales.Vibrionaceae**	D-UVR+	3.85	0.0343
**Bacteria.Proteobacteria.Gammaproteobacteria.Vibrionales.Vibrionaceae.Photobacterium**	D-UVR+	3.82	0.0186
**Bacteria.Proteobacteria.Gammaproteobacteria.Vibrionales.Vibrionaceae.Photobacterium.OTU216**	D-UVR+	3.82	0.0186
**Bacteria.Bacteroidetes.Bacteroidia.Bacteroidales.Rikenellaceae.Rikenellaceae_unclassified.OTU54**	D+UVR-	4.11	0.008
**Bacteria.Bacteroidetes.Bacteroidia.Bacteroidales.S24_7**	D+UVR-	4.49	0.047
**Bacteria.Bacteroidetes.Bacteroidia.Bacteroidales.S24_7.S24_7_unclassified**	D+UVR-	4.49	0.047
**Bacteria.Bacteroidetes.Bacteroidia.Bacteroidales.S24_7.S24_7_unclassified.OTU58**	D+UVR-	4.49	0.047
**Bacteria.Verrucomicrobia**	D+UVR-	4.87	0.0283
**Bacteria.Verrucomicrobia.Verrucomicrobiae**	D+UVR-	4.87	0.0283
**Bacteria.Verrucomicrobia.Verrucomicrobiae.Verrucomicrobiales**	D+UVR-	4.87	0.0283
**Bacteria.Verrucomicrobia.Verrucomicrobiae.Verrucomicrobiales.Verrucomicrobiaceae**	D+UVR-	4.87	0.0283
**Bacteria.Verrucomicrobia.Verrucomicrobiae.Verrucomicrobiales.Verrucomicrobiaceae.Akkermansia**	D+UVR-	4.87	0.0283
**Bacteria.Verrucomicrobia.Verrucomicrobiae.Verrucomicrobiales.Verrucomicrobiaceae.Akkermansia.OTU239**	D+UVR-	4.87	0.0283

Threshold used was linear discriminant analysis (LDA) score > 2 and $P < 0.05$. The results provide a list of bacterial taxa at all taxonomic levels that are differentially abundant between the D−UVR+ and D+UVR− groups at Day 35.
